# EBUS-TBNA Provides Highest RNA Yield for Multiple Biomarker Testing from Routinely Obtained Small Biopsies in Non-Small Cell Lung Cancer Patients - A Comparative Study of Three Different Minimal Invasive Sampling Methods

**DOI:** 10.1371/journal.pone.0077948

**Published:** 2013-10-29

**Authors:** Gerald Schmid-Bindert, Yongsheng Wang, Hongbin Jiang, Hui Sun, Thomas Henzler, Hao Wang, Lothar R. Pilz, Shengxiang Ren, Caicun Zhou

**Affiliations:** 1 Department of Surgery, University Medical Center Mannheim, Medical Faculty Mannheim of Heidelberg University, Mannheim, Germany; 2 Department of Medical Oncology, Shanghai Pulmonary Hospital, Tongji University School of Medicine, Tongji University Medical School Cancer Institute, Shanghai, China; 3 Department of Respiratory Medicine, Nanjing Drum Tower Hospital, Nanjing University Medical School, Nanjing, China; 4 Emergency Department, Shanghai Pulmonary Hospital, Tongji University School of Medicine, Shanghai, China; 5 Institute of Clinical Radiology and Nuclear Medicine, University Medical Center Mannheim, Medical Faculty Mannheim of Heidelberg University, Mannheim, Germany; 6 Department of Surgery, Shanghai Pulmonary Hospital, Tongji University School of Medicine, Shanghai, China; 7 Medical Faculty, Mannheim of Heidelberg University, Mannheim, Germany; Univesity of Texas Southwestern Medical Center at Dallas, United States of America

## Abstract

**Background:**

Multiple biomarker testing is necessary to facilitate individualized treatment of lung cancer patients. More than 80% of lung cancers are diagnosed based on very small tumor samples. Often there is not enough tissue for molecular analysis. We compared three minimal invasive sampling methods with respect to RNA quantity for molecular testing.

**Methods:**

106 small biopsies were prospectively collected by three different methods forceps biopsy, endobronchial ultrasound (EBUS) guided transbronchial needle aspiration (TBNA), and CT-guided core biopsy. Samples were split into two halves. One part was formalin fixed and paraffin embedded for standard pathological evaluation. The other part was put in RNAlater for immediate RNA/DNA extraction. If the pathologist confirmed the diagnosis of non-small cell lung cancer(NSCLC), the following molecular markers were tested: EGFR mutation, ERCC1, RRM1 and BRCA1.

**Results:**

Overall, RNA-extraction was possible in 101 out of 106 patients (95.3%). We found 49% adenocarcinomas, 38% squamouscarcinomas, and 14% non-otherwise-specified(NOS). The highest RNA yield came from endobronchial ultrasound guided needle aspiration, which was significantly higher than bronchoscopy (37.74±41.09 vs. 13.74±15.53 ng respectively, P = 0.005) and numerically higher than CT-core biopsy (37.74±41.09 vs. 28.72±44.27 ng respectively, P = 0.244). EGFR mutation testing was feasible in 100% of evaluable patients and its incidence was 40.8%, 7.9% and 14.3% in adenocarcinomas, squamouscarcinomas and NSCLC NOS subgroup respectively. There was no difference in the feasibility of molecular testing between the three sampling methods with feasibility rates for ERCC1, RRM1 and BRCA1 of 91%, 87% and 81% respectively.

**Conclusion:**

All three methods can provide sufficient tumor material for multiple biomarkers testing from routinely obtained small biopsies in lung cancer patients. In our study EBUS guided needle aspiration provided the highest amount of tumor RNA compared to bronchoscopy or CT guided core biopsy. Thus EBUS should be considered as an acceptable option for tissue acquisition for molecular testing.

## Introduction

Lung cancer is the leading cause of cancer related mortality worldwide with >1.3 million estimated deaths in 2008 [1]. Non-small cell lung cancer (NSCLC) accounts for more than 80% of newly diagnosed cases, and most patients are diagnosed with advanced stage disease. The individualization of treatment with new cytotoxic agents and targeted therapies, such as pemetrexed, bevacizumab, gefitinib, erlotinib or crizotinib, has made it crucial now to further subclassify NSCLC by histological and molecular criteria [2]–[5].

However, more then 80% of patients are diagnosed on the basis of very small biopsies or cytology samples [6]. While the tumor tissue for multiple biomarker testing is permanently increasing on one hand, the size of tissue samples on the other hand is rather decreasing with the advent of new minimal invasive diagnostic tools such as endobronchial ultrasound guided needle aspiration (EBUS-TBNA). Given the small sample sizes obtained by routine lung cancer diagnostic procedures, tissue may already be expended after histopathological evaluation of the tumor and testing for EGFR mutation. Cytological samples obtained by EBUS have often been claimed as insufficient for molecular testing, especially in clinical trials and for research purposes.

The main sources for tissue in advanced lung cancers are bronchoscopic forceps biopsies, CT-guided core biopsies and EBUS-TBNA. The aim of this study was to compare these three sampling methods with respect to the yield of extractable RNA for molecular testing in routinely performed diagnostic procedures of lung cancer patients. To avoid various degrees of RNA degradation by the process of fixation and paraffin embedding, we used a method for tissue banking of diagnostic lung cancer biopsies recently reported by Lawson et al. [7]. This method provides RNA of better quality compared to fresh frozen tissue and can be applied very easily in a routine clinical setting.

## Materials and Methods

This study was conducted at Shanghai Pulmonary hospital, Tongji University Shanghai, China in a bilateral cooperation project with Medical Center Mannheim of Heidelberg University. We prospectively screened all patients that were suspicious for lung cancer due to clinical and radiological evidence. Depending on tumor localization, the responsible physician performed bronchoscopy, CT-guided core biopsy or EBUS-TBNA for histological or cytological confirmation of lung cancer.

### Patients

From October 2010 to September 2011, 106 patients who have been diagnosed as NSCLC were considered eligible and enrolled in this study. Five patients were assessed as not evaluable because of insufficient material. All patients with confirmed NSCLC from histological or cytological samples by experienced pathologists were included into this analysis. The histologic diagnosis was based on the World Health Organization classification [8]. The protocol was conducted according to the Declaration of Helsinki and Good Clinical Practice guidelines and was approved by the ethics committee of Tongji University Affiliated Shanghai Pulmonary Hospital. The informed consent was written and obtained from all patients before the initiation of this study.

### Sample Collection

Biopsies were taken either by endobronchial biopsy forceps (Olympus Endojaw Single-use biopsy forceps;) via fiberoptic bronchoscope (Olympus BF-6C260), by endobronchial ultrasound guided transbronchial needle aspiration (EBUS-TBNA) (Olympus BF-UC260F-OL8; 22-gauge needles, Olympus NA-2015X-4022) or by computed tomography-guided needle core biopsy (16–20-gauge Quick-Core Biopsy Needle). Which of these three methods was used for tissue sampling was based on the location of the primary tumor and mediastinal or hilar lymph node status. Once the operator had obtained adequate tissue, biopsies were split into two equivalent parts. Half of the biopsy was sent to pathology. The other half of the biopsy was immediately preserved in RNAlater, which means that samples were placed in an Rnase free Eppendorf tube containing 500 ul of RNAlater (at least ∼10 times the sample volume), and stored at 4°C. If the pathologist confirmed the first half of the sample as NSCLC, then the stored samples were taken out to extract RNA and DNA. In short, the samples were taken out by forceps in RNAlater and cut into pieces on a clean surface. Then, they were transferred into tubes and were processed as described in the handbook. All extracted RNA was quantified. If no diagnosis of malignancy was obtained from the diagnostic biopsies, the study biopsies were removed. Thus, only samples with confirmed NSCLC were stored for further analysis.

We used the Allprep DNA/RNA mini kit (Qiagen, Germany) to extract and purify the RNA and DNA simultaneously. It is a technique of collecting data throughout the PCR process as it occurs, thus combining amplification and detection into a single step. RT-PCR even needs less RNA to run the gene expression analysis. In our study, we performed the RNA quantification and quality analysis with Nanodrop-2000. RNA and DNA were stored at −80°C for future biomarker analysis.

### ERCC1, RRM1 and BRCA1 mRNA Analysis

All samples were analyzed for mRNA levels of ERCC1, RRM1, and BRCA1. For cDNA synthesis, 2 ul of RNA was preserved in 70°C for 10 min, then added 4 ul of 25 mmol/L MgCl_2_, 2 ul of 10×reverse transcriptase buffer, 2 ul of 10 mmol/L dNTP, 0.5 ul of RNAse inhibitor, 0. 5 Ug of Oligo (dT) 15, 15 U of AMV reverse transcriptase, filled up to a total volume of 20 uL. Reverse transcription reaction was undergone in a temperature of 42°C for 15 minutes. Then, relative cDNA quantitation for ERCC1, RRM1, and BRCA1 was determined using a fluorescent, real-time detection method (Light 2 Cycler 2. 0, Roche Company) with an internal reference gene (β-actin) as control.

Amplification was carried out in a total volume of 25 µl containing 0.25 µmol/L of each primer, 0.02 mmol/L dNTPs and 1 mmol/L MgCl2, 1.25 U Taq polymerase and 5×PCR buffer. The PCR program initiated with 1 min denaturation at 95°C. The DNA was amplified by one cycle of 95°C for 5 s and 50 cycles of 92°C for 40 s, followed by elongation at 60°C for 40 s. The details were described in our previous publications [5], [9]–[10]. The gene expression analysis was performed in a blinded fashion where the laboratory investigators were unaware of the clinical data.

### EGFR Mutation Analysis

A method for rapid detection of EGFR mutation types in 80 resected NSCLC tissues with PCR combined with Taqman probes was previously reported and described in detail there [11]. This method is able to detect EGFR mutations, when samples contain 10% tumor, and at least 50 tumor cells are necessary for a positive test. Only mutations in exon 19 and 21 are detected by this method.

### Statistical Analysis

Standard descriptive statistical analysis for the data was performed. Smoking status was reevaluted into never smokers, and smokers. Tumor stage was classified into advanced (IIIB and IV) and not advanced (IA–IIIA) stage. Testing of groups was done with the two-sided Wilcoxon rank-sum test and in case of multiple testing the Bonferroni-Holms correction for the total significance level was used. Significance level was set to α = 0.05. For descriptive statistical analysis StatXact-9 of Cytel Studio, Version 9.0.0, Cytel Inc., Cambridge, MA, USA and for the tests the SAS software 9.2 (TS2M3) by the SAS Institute Inc., Cary, NC, USA were used.

## Results

### Patients and Material

A total of 106 patients with confirmed NSCLC were enrolled into this study, 101 samples were suitable for RNA extraction. Patient characteristics including age, gender, smoking status and tumor related data such as stage, histology and RNA quantity measured in ng/Ul are shown in table 1. From the drawn samples multiple biomarker analyses were performed: EGFR-mutation, ERCC1, RRM1, and BRCA1. The patients enrolled in the study represent the typical lung cancer population, with a median age of 61 (38–79) years, a majority being male (76%) and current or former smokers (61%). Histology could be subclassified into adenocarcinoma (49%) and squamous cell carcinoma (38%), while 14% were classified as NSCLC-NOS. 25.7% of patient were diagnosed in early tumor stage I (12.4%) or II (13.3%). 25 were stage III (25.7) most common was stage IV (48.6%). The sampling methods were bronchoscopy in 45 cases (44.6%), EBUS-TBNA in 33 cases (32.7%) and CT core biopsy in 23 cases (22.8%).

**Table 1:** Patient and tumor characteristics of 101 evaluable patients.

Characteristics	All patients (N = 101)	Bronchoscopy (N = 45)	EBUS-TBNA (N = 33)	CT Core Biopsy (N = 23)
Age				
>65	33(32.7%)	33(73.3%)	17(51.5%)	18(78.3%)
≤65	68(67.3)	12(26.7%)	16(48.5%)	5(21.7%)
Gender				
Male	77 (76%)	37 (82%)	27 (82%)	13 (57%)
Female	24 (24%)	8 (18%)	6 (18%)	10 (43%)
Smoking status				
Smoker	62 (61%)	30 (67%)	23 (70%)	9 (39%)
Never smoker	39 (39%)	15 (33%)	10 (30%)	14 (61%)
ECOG PS				
0–1	79(78.2%)	35(77.8%)	24(72.7%)	20(87.0%)
2	22(21.8%)	10(22.2%)	9(27.3%)	3(13.0%)
Stage				
Stage I–IIIa	31 (31%)	18 (40%)	6 (18%)	7 (30%)
Stage IIIb–IV	67 (66%)	25 (56%)	26 (79%)	16 (70%)
Not evaluated	3 (3%)	2 (4%)	1 (3%)	0 (0%)
Histology				
Adeno C	49 (49%)	17 (38%)	15 (45%)	17 (74%)
Squamous CC	38 (38%)	23 (51%)	10 (30%)	5 (22%)
NOS	14 (14%)	5 (11%)	8 (24%)	1 (4%)
EGFR mutations				
Activated	25 (24.8%)	8 (17.8%)	10 (31.3%)	7 (30.4%)
Wild type	76 (75.2%)	37 (82.2%)	22 (68.8%)	16 (69.6%)

### RNA Quantity

**Figure 1:**
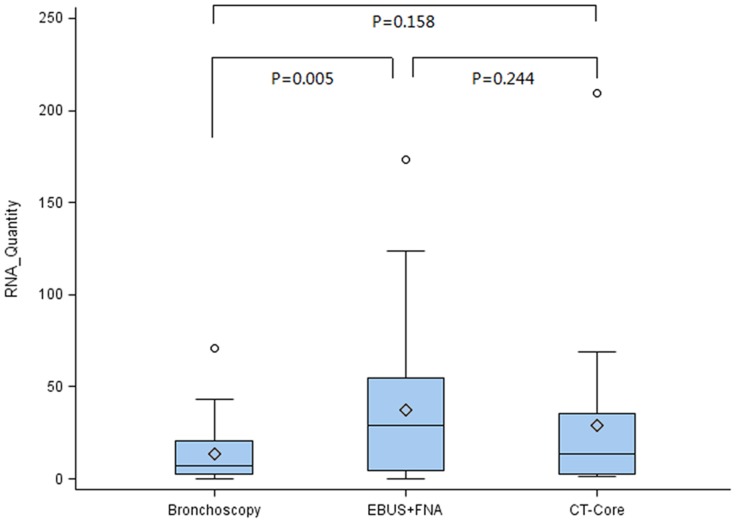
RNA-quantity was 13.74±15.53 ng, 37.74±41.09 ng and 28.72±44.27 ng in the bronchoscopy group, EBUS TBNA group and CT core biopsy group respectively. Among them, Tumor RNA-quantity in EBUS TBNA group was significantly higher than in the bronchoscopy group (P = 0.005), while no statistical significance existed between CT-core biopsy and either of the other two methods.

For all evaluable tumor samples (n = 101), the RNA content was measured with a mean of 24.99 ng/Ul, ranging from 0.01 to 209.20 with a standard deviation of 34.52 (figure 1 and table 2). The RNA- yield was compared between the sampling methods. The highest amount of tumor RNA was obtained by EBUS-TBNA with a median of 28.9 ng/UL (0.3–173.1 ng/UL); the lowest yield was reached by bronchoscopy with a median of 7.2 ng/UL (0.01–70.6 ng/UL). The difference between the two methods was statistically significant (P = 0.005). The amount of tumor-RNA obtained by CT-core biopsy was somewhere between the two other methods with a median of 13.8 ng/UL (1.2–209 ng/UL), and no significant difference was seen between CT-core biopsy and either of the other two methods (figure 1).

**Table 2:** RNA quantity measurements in three different sampling methods, RNA quantity is given in ng.

Specimen	Mean	Median	StdDev	Variance	Min	Max
Bronchoscopy(n = 45)	13.74	7.2	15.53	241.3	0.01	70.6
EBUS-TBNA(n = 33)	37.74	28.9	41.09	1688	0.3	173.1
CT Core biopsy(n = 23)	28.72	13.8	44.27	1960	1.2	209.2
All patients(n = 101)	24.99	10.2	34.52	1191	0.01	209.2

StdDev = Standard deviation.

### EGFR Mutation Analysis

In 101 evaluable samples, we detected 25 samples with EGFR mutation (table 1), 20 out of 25 were adenocarcinoma. The rate of detected EGFR mutation in the adenocarcinoma subgroup (n = 49) was 40.8%, which was significantly higher than 7.9% in the patients with squamous cell carcinomas and 14.3% in the NSCLC NOS. Also, the incidence of activated EGFR mutation was more likely to happen in female patients (54.2% vs 15.7%, P<0.001) and never smokers (35.9% vs 17.7%, P = 0.04).

### ERCC1, RRM1 and BRCA1 mRNA Quantity

Results of the three measured biomarkers ERCC1, RRM1 and BRCA1 mRNA Quantity are shown in table 3. Biomarker results were available for 80% to 91% of samples. In the subgroup analysis, there were no significant differences of the ERCC1, RRM1 and BRCA1 mRNA Quantity in different histology types such as adenocarcinoma, squamous cell carcinoma and not otherwise specified carcinomas (multiple Wilcoxon rank-sum test) (table 4). However, when we investigated the association of EGFR mutation and these three mRNA levels, we found that ERCC1 mRNA level was significantly lower in those patients with activated EGFR mutation than in wild type (P = 0.013), whereas RRM1 or BRCA1 mRNA levels were not related to EGFR mutation status (table 5).

**Table 3:** Overall results of measurements of all mRNA markers.

Items	Mean	Median	StdDev	Variance	Min	Max
ERCC1(n = 92)	0.1195	0.0104	0.6727	0.4526	0.0001	5.5439
RRM1 (n = 88)	0.1948	0.0214	1.1106	1.2330	0.0001	10.1999
BRCA1 (n = 81)	0.2657	0.0017	1.3671	1.8690	0.0001	10.6894

StdDev = Standard deviation.

**Table 4:** Measurements of variables per patient by histology.

Items	Mean	Median	StdDev	Variance	Min	Max
ERCC1(n = 92)						
Adeno (n = 45)	0.1497	0.0104	0.8259	0.6821	0.0001	5.5439
Squamous (n = 34)	0.1171	0.0118	0.5772	0.3332	0.0004	3.3821
Nos (n = 13)	0.0214	0.0083	0.0322	0.0010	0.0001	0.1072
RRM1 (n = 88)						
Adeno (n = 43)	0.0294	0.0187	0.0303	0.0009	0.0001	0.1578
Squamous (n = 33)	0.1602	0.0291	0.4139	0.1721	0.0001	2.0499
Nos (n = 12)	0.8695	0.0133	2.9184	8.6344	0.0003	10.1999
BRCA1(n = 81)						
Adeno (n = 42)	0.2064	0.0016	0.9238	0.853	0.0001	4.8213
Squamous (n = 29)	0.0713	0.0016	0.3737	0.1397	0.0001	2.0499
Nos (n = 10)	1.0716	0.0034	3.3794	11.4201	0.0003	10.6894

StdDev = Standard deviation.

**Table 5:** Measurements of variables per patient by mutation status.

Items	Mean	Median	StdDev	Variance	Min	Max	P
ERCC1 (n = 92)							0.013
EGFR mutpos. (n = 23)	0.0666	0.0102	.4057	0.1645	0.0001	3.3815	
EGFR mutneg. (n = 69)	0.2783	0.0118	1.1525	1.3280	0.0003	5.5439	
RRM1 (n = 88)							0.143
EGFR mutpos. (n = 22)	0.0261	0.0186	0.0247	0.001	0.0003	0.0972	
EGFR mutneg. (n = 66)	0.2510	0.0240	1.2798	1.638	0.0001	10.1999	
BRCA1(n = 81)							0.800
EGFR mutpos. (n = 21)	0.2337	0.0015	1.0512	1.105	0.0001	4.8213	
EGFR mutneg. (n = 60)	0.2769	0.0022	1.4694	2.159	0.0001	10.6894	

StdDev = Standard deviation.

## Discussion

In the present study we prospectively collected small biopsy samples from a relatively large cohort of untreated patients with newly diagnosed NSCLC to immediately extract RNA and DNA for molecular analysis. Our results demonstrate that all three minimal-invasive methods for tissue sampling - CT-core biopsy, bronchoscopy, EBUS - can provide sufficient material for additional biomarker testing in most cases. It has been reported before, that testing for single molecular markers such as EGFR-mutation from very small tissue samples obtained by EBUS is feasible [12]–[15]. Furthermore, Billah et al also showed that it was feasible to test two mutations (EGFR and KRAS) in cytology specimen such as EBUS and CT guided FNAs [16]. However, in most of these reports, patient numbers were quite small and only one or two markers were tested in each sample. In the future we will have to test for many more molecular markers as a standard of care in the treatment of patients with lung cancer. Moreover, in the field of clinical research, cytology specimens derived from EBUS are still not accepted for mutation analysis if tissue is mandatory to include a patient into a clinical trial. Overall, we have to find new ways to extract enough RNA for multiple biomarker testing, for clinical routine as well as research purposes, and it remains unclear how the increasing number of molecular markers, which have to be tested for an individualized treatment stragety of NSCLC could be analyzed from routinely obtained small biopsy samples, especially from EBUS-TBNA.

As an objective measure method for the suitability of sampling, we defined the amount of tumor RNA extractable for biomarker analysis. In our study we adopted the method described by Lawson et al. [7], where half of the biopsy samples were collected immediately for RNA extraction without prior formalin fixation and paraffin embedding. This method has the advantage that one part of the tissue can be processed by the pathologist for histopathological diagnosis, while at the same time the other part can be preserved for RNA-extraction and PCR examination. From 101 out of 106 patients, we successfully collected tissue by bronchoscopy (n = 45), EBUS (n = 33) and CT-guided core biopsy (n = 23) suitable for RNA extraction. EGFR mutation testing was evaluable in all cases. There was no difference in the feasibility of molecular testing between the three sampling methods. Overall feasibility for ERCC1, RRM1 and BRCA1 was 91%, 87% and 81%, respectively.

We found 24.8% of EGFR mutations in our enrolled Asian population, the frequency of EGFR mutations seemed a little lower then expected [17]–[19]. However, most of our patients were male, smokers and non-adenocarcinomas, which might be the main reason for the lower incidence of the activated EGFR mutation. In the subpopulation of adenocarcinomas the rate of EGFR mutations was 40.8%. Apart from that, the method used for mutation testing in our study needed 50 tumor cells for a positive test result, and a least 10% of mutated cells within the cell population. Also, we only detected the main EGFR mutations in exon 19 and 21. Thus, if the amount of mutated tumor cells in the sample is below that limit or some other uncommon EGFR mutation was present, the test would be false negative. Results of ERCC1, RRM1, and BRCA1 showed a remarkable variability, as also reported previously [20]. Consistent with findings from other studies, median ERCC1 levels tended to be lower in adenocarcinoma (median 0.0104) than in squamous carcinoma (median 0.0118; n.s.). The same was observed for RMM1 (median 0.0187 and 0.0291, respectively). Thus the obtained biomarker results are in concordance with previous reports and demonstrate that the used method is feasible for multiple biomarker testing in a routine setting. In line with our previous and Gandara’s report [20]–[21], the ERCC1 levels in the patients with EGFR mutation were significantly lower than the wild type, which indicated that EGFR mutation could also be helpful as a selection criterion for the optimal chemotherapy regimen.

The main goal of the study was to compare the different techniques used for tissue sampling. Interestingly, EBUS was the best method for securing high amounts of tumor RNA. The content of tumor RNA obtained by EBUS-TBNA was significantly higher compared to bronchoscopy (37.74±41.09 ng versus 13.74±15.53 ng, respectively; p = 0.005). These findings may be explicable by the known differences of the used methods: bronchoscopic forceps biopsies are taken from tumor periphery. Distinction between necrosis, inflammatory mucosa and vital tumor is not easy through bronchoscopy. Moreover, repeated biopsies may result in bleeding, which often leads to preliminary discontinuation of the procedure without having achieved the optimal tissue yield. CT guided core biopsy may cause pneumothorax, so the procedure will mostly be done only one time to reduce risk of complication and thus only limited amount of tissue is available. EBUS-TBNA on the other hand is a very safe method with a complication rate near to zero [22], thus repeated needle aspirations for higher diagnostic yields are possible without compromising the patient. Moreover, needle aspirations are taken under direct vision and by using power Doppler imaging and general morphological ultrasound criteria, thus necrotic areas of lymph nodes can be avoided for biopsy most of the time [23].

In summary, our study confirms that all three different minimal invasive techniques can provide sufficient material for molecular analysis in most of the patients. After testing for the four different markers, there was still RNA left for further analysis of more markers. EBUS-TBNA is a very safe method with almost no risk of complication and has achieved the highest yield of tumor RNA in our study. Therefore it should be an acceptable option for tissue sampling in the era of personalized oncology.
